# Organelle Cross-Talk in Apoptotic and Survival Pathways

**DOI:** 10.1155/2012/968586

**Published:** 2012-12-25

**Authors:** Lina Ghibelli, Alina Grzanka

**Affiliations:** ^1^Dipartimento di Biologia, Universita' di Roma Tor Vergata, via della Ricerca Scientifica, 100133 Roma, Italy; ^2^Department of Histology and Embryology, Nicolaus Copernicus University, Bydgoszcz, Poland

Apoptosis and survival pathways are intimately connected signal transduction cascades that determine the fate of cells suffering from insults such as DNA damage, oxidative stress, intracellular Ca^2+^ overload, mild excitotoxicity, accumulation of unfolded or misfolded proteins in the endoplasmic reticulum (ER), and many others, or induced to autoelimination by extracellular signals. The apoptotic pathways, respectively, intrinsic or extrinsic, are executed through molecular signals travelling inside the cells. If the initial studies considered the nucleus, and specifically DNA, as both primary sensor and executioner of apoptosis, active roles of other intracellular organelles have been pointed out in the last two decades, identifying plasma membrane as the initiator of the extrinsic pathway and mitochondria as the pivot of the intrinsic pathway. In apoptosing cells, the outer membrane of mitochondria is permeabilized by the proapoptotic members of the Bcl-2 family, promoting the release of proapoptotic factors such as the primary and secondary activators of caspases (cytochrome c and SMAC/diablo, resp.). Stress signals, typically inducing the intrinsic apoptotic pathway, must be perceived by the damaged cells and translated into signals that promote mitochondria permeabilization, after which caspases are activated. This implies that other organelles participate in apoptotic signaling upstream to mitochondria involvement, acting as sensors of damage or signal transducers. Recently, a key role of the endoplasmic reticulum (ER) is emerging, consisting of the ability of amplifying or dampening the apoptotic signal prior to mitochondria involvement, through the establishment of active Ca^2+^ fluxes that control the physical and functional interaction between mitochondria and ER. Also lysosomes participate to the intrinsic apoptotic signaling, and their dialogs with mitochondria have been described. Other important examples of organelles cross-talk in apoptosis include nucleus-mitochondria exchange of proteins such as p53, which is main controller of the cell response to DNA damage. To date, the role of organelles other than mitochondria in perceiving and signaling cell damage, and the interrelation of the organelles during apoptotic signaling, are still poorly explored matters.

In this special issue dedicated to Organelle Cross-talk in Apoptotic and Survival Pathways, original research papers and review articles are collected with the aim of giving an impulse to this promising field of research on apoptosis and stimulating discussion between interdisciplinary readers.

The paper written by Y.-B. Ouyang and R. G. Giffard describes ER-mitochondria cross-talk during cerebral ischemia. The authors precisely describe the chaperone machineries at both the ER and mitochondrion. Moreover, they contribute the new view that the chaperones organise not only the regulation of Ca^2+^ signaling between these two organelles and control bioenergetics, cell survival, but also cell death decision.

In the paper entitled “*Mitochondrial dynamics: functional link with apoptosis*”, written by H. Otera and K. Mihara, the readers are introduced to “intrinsic” apoptotic pathway in the context of regulation and physiologic significance of mitochondrial fusion and fission. The authors of this review underline also that the ER-mitochondria contact is involved in the regulation of mitochondrial energy, lipid metabolism, calcium signaling, and even in mitochondrial fission.

An important issue arising is the cross-talk of apoptosis with autophagy, a stress response pathway controlled by key intracellular regulators such as m-TOR, induced by insults or conditions ranging from nutrient depletion, protein malfolding, or cell ageing. The paper written by H. Rikiishi focusing on the recent advances regarding autophagy in cancer and the techniques currently available to manipulate autophagy. The author of this paper provides a complete overview of the literature on molecular mechanism of autophagy and its cross-talk with apoptosis. Additionally, the author indicates the technical problems in the study of autophagy and suggest that new methods need to be developed to ensure progress in this area of investigation. Moreover, they highlight that the multifaceted nature of autophagy and its diverse cross-talk with other biological processes must be carefully considered when the autophagic system is targeted for anticancer benefits. 

The paper entitled “*Effects of cisplatin in neuroblastoma rat cells: damage to cellular organelles*” written by G. Santin et al. provides the practical analysis of organelle cross-talk during cell death. The authors indicate that B50 neuroblastoma cells treated with cisplatin undergo apoptosis through organelles injury, and not only as a consequent of DNA damage, but also that cisplatin does not induce any autophagic process as alternative response to the treatment.

In conclusion, the studies collected in this issue effectively contribute to the general problem of organelles as target of cell damage versus transducer of the apoptotic signal.

*Lina Ghibelli* *Lina Ghibelli* *Alina Grzanka* *Alina Grzanka*

